# Supplementary material for: The prevalence and correlates of depression among patients with chronic diseases in the United Arab Emirates
Source: PLoS One. 2022 Dec 14;17(12):e0278818. doi: 10.1371/journal.pone.0278818 (PMC9749973; doi:10.1371/journal.pone.0278818)
Supplement: Attachment — Submitted filename: Response to Reviewers.docx [file pone.0278818.s001.docx]

**Response to Reviewers Comments**

We express our gratitude to both reviewers for taking their time to review our manuscript and provide valuable feedback that has significantly improved our manuscript. We have endeavored to revise our manuscript thoroughly in line with reviewers’ suggestions. To this end, we have provided detailed responses to each comment raised by the reviewers and have mentioned the page and line numbers of changes in the manuscript. We have also incorporated reviewers’ suggestions and submitted the revised manuscript as cleaned and tracked versions.

**Reviewer #1 comment:**

1. *I would suggest to scale the y-axis of figure 1 from 0-100.*

**Action:** Figure 1 y-axis has been rescaled to 0-100

**Reviewer #2 (Dr. Stewart Ndutard Ngasa) comments:**

**Comment 1**

*The article is entitled 'The Prevalence and Correlates of Depression among Patients with Chronic Diseases in the United Arab Emirates'. This suggests that all participants recruited have a chronic medical condition. In the manuscript, I couldn't find anywhere the definition for what was considered a chronic medical condition and how this guided the authors in choosing their participants. I would suggest including a section for inclusion and exclusion criteria. This will make it clearer for the readers.*

**Authors’ response:**

1- Thank you for the reviewer for pointing out to include a section on inclusion and exclusion criteria and a definition of a chronic medical condition.

**Actions:**

We have included the definition in the introduction (Page: 4, Line: 100-103). Although we had an inclusion under the section of participants and sample size line 145 we have elaborated more in that section on inclusion criteria. (Page:6, Line :147-148)

**Comment 2**

*2. I also found the analysis presented in table 2 a bit confusing, can the authors confirm that everyone who was included in the study had a chronic medical condition? If that was the case then our input variable can be broadly classified into 'present or absent' of a chronic medical condition and the outcome variables will be the correlates or associated factors. A separate table may be used to show the prevalence of depression in the different categories of chronic medical condition present in the participants*

**Authors’ response:**

2- Yes, all participants in the study had a chronic medical condition.

In Table 2 we studied the association between depression severity (outcome variable) and different demographic and comorbidities characteristics (covariate/factor variables). In this table, categorical variables were analyzed using Chi-square or Fisher’s exact test and continuous variable by Kruskal-Wallis test. Represents a medical condition (e.g. Type I diabetes) with one row (Present or Absent) instead of two rows (No & Yes) will only reduce the number of rows (i.e. table size) since eventually, we will use the Chi-square or Fisher’s exact test to get a p-value to decide if there is a significant association or not between the medical condition and depression severity.

3- We thank the reviewer for this suggestion.

**Actions:**

1- To improve the understanding of Table 2. We restructured and moved the text describing Table 2 into a separate paragraph, updated the table’s title and added a footnote (Page: 9, Line: 216-220).
2- We added Fig 2 with the prevalence of depression in selected chronic diseases; diseases with adequate representation in the sample to provide an acceptable estimation of prevalence (Page: 8, Line: 206-207).

**Comment 3**

*3. The authors have suggested in the paragraph following table 2 that the authors are describing a univariate result analysis. This seems to be the results of a bivariate analysis with Crude Odds Ratios presented. Can the authors verify this.*

**Authors’ response**

We carried out a *univariate logistic regression analysis,* in which we examined the association of different characteristics with the presence of depression separately, this means that we fitted many models in each model we had one variable (e.g. age) and the outcome variable (depression). Please note that Tsai in his paper “*Achieving consensus on terminology describing multivariable analyses*” stated that a regression model studying the effect of single explanatory variable on the response variable is a univariable analysis. Using the term “bivariate” to describe such a model, while common, introduces unnecessary confusion [1].

**Action:** To avoid confusion, we replaced univariate and multivariable words in the manuscript with unadjusted/simple and adjusted/multiple logistic regression analysis, respectively (Page: 7, Line: 192, 196, 198; Page: 11, Line: 225, 230).

**Comment 4**

*4. I was wondering if there was any rational for choosing the Ambulatory Health Services for the recruitment of patients, knowing that patients who present to such services usually come in an acute state. This might influence the way participants respond to the questions on the PHQ questionnaire. Recruiting patients in a more stable environment might give a totally different results.*

**Authors’ response:**

*We agree with the reviewer. However, we chose it as the AHS clinics as those clinics provide the service of diagnosis, treatment, and follow-up with patients with chronic diseases. They have a special clinic for patients with chronic diseases (called CDC clinic). The choice was based on easy access to these patients and to raise awareness among the AHS staffs about the need to follow up on mental health of these patients. I would like to point that all patients receive the PHQ9 as part of the assessment, however, there are no data currently on the prevalence and the needs of mental health support for these patients.*

**Actions:**

We have added an explanation in (Page:5, Line:132-140) for the choice of AHS

References

[1] Tsai AC. Achieving consensus on terminology describing multivariable analyses. Am J Public Health. 2013 Jun;103(6):e1. doi: 10.2105/AJPH.2013.301234. Epub 2013 Apr 18. PMID: 23597350; PMCID: PMC3679183.

**Editors Comments and Journal Requirements:**

When submitting your revision, we need you to address these additional requirements.

1. Please ensure that your manuscript meets PLOS ONE's style requirements, including those for file naming. The PLOS ONE style templates can be found at
   https://journals.plos.org/plosone/s/file?id=wjVg/PLOSOne_formatting_sample_main_body.pdf and 
   <https://journals.plos.org/plosone/s/file?id=ba62/PLOSOne_formatting_sample_title_authors_affiliations.pdf>

**Response: Please note that the manuscript was edited as per journal requirement**

2. Please provide additional details regarding participant consent. In the ethics statement in the Methods and online submission information, please ensure that you have specified what type you obtained (for instance, written or verbal, and if verbal, how it was documented and witnessed). If your study included minors, state whether you obtained consent from parents or guardians. If the need for consent was waived by the ethics committee, please include this information.

**Response: Please note that a statement on the consent was added in page 6, line 162-166**

3. We note that the grant information you provided in the ‘Funding Information’ and ‘Financial Disclosure’ sections do not match. 
When you resubmit, please ensure that you provide the correct grant numbers for the awards you received for your study in the ‘Funding Information’ section.

**Response: We thank the editor for pointing out this issue.**

This study was funded by SUREPLUS Grant, G00003183 from United Arab Emirates University to be added in funding information

4. Thank you for stating the following in the Acknowledgments Section of your manuscript: 
"This study was funded by SUREPLUS Grant, G00003183 from United Arab Emirates University."

We note that you have provided funding information that is not currently declared in your Funding Statement. However, funding information should not appear in the Acknowledgments section or other areas of your manuscript. We will only publish funding information present in the Funding Statement section of the online submission form.

Please remove any funding-related text from the manuscript and let us know how you would like to update your Funding Statement. Currently, your Funding Statement reads as follows:

"This study was funded by SUREPLUS Grant, G00003183 from United Arab Emirates University. The funders had no role in study design, data collection and analysis, decision to publish, or preparation of the manuscript."

Please include your amended statements within your cover letter; we will change the online submission form on your behalf.

**Response: Please note that we removed information on funding from manuscript. Please add the following statement to the funding information**

***"This study was funded by SUREPLUS Grant, G00003183 from United Arab Emirates University. The funders had no role in study design, data collection and analysis, decision to publish, or preparation of the manuscript."***

5. In your Data Availability statement, you have not specified where the minimal data set underlying the results described in your manuscript can be found. PLOS defines a study's minimal data set as the underlying data used to reach the conclusions drawn in the manuscript and any additional data required to replicate the reported study findings in their entirety. All PLOS journals require that the minimal data set be made fully available. For more information about our data policy, please see http://journals.plos.org/plosone/s/data-availability.

Upon re-submitting your revised manuscript, please upload your study’s minimal underlying data set as either Supporting Information files or to a stable, public repository and include the relevant URLs, DOIs, or accession numbers within your revised cover letter. For a list of acceptable repositories, please see http://journals.plos.org/plosone/s/data-availability#loc-recommended-repositories. Any potentially identifying patient information must be fully anonymized.

Important: If there are ethical or legal restrictions to sharing your data publicly, please explain these restrictions in detail. Please see our guidelines for more information on what we consider unacceptable restrictions to publicly sharing data: http://journals.plos.org/plosone/s/data-availability#loc-unacceptable-data-access-restrictions. Note that it is not acceptable for the authors to be the sole named individuals responsible for ensuring data access.

We will update your Data Availability statement to reflect the information you provide in your cover letter.

**Response: we have uploaded the anonymized data set to a public repository Harvard Dataverse, available at the following DOI** <https://doi.org/10.7910/DVN/NRJNEN>.
